# Auditory Manifestations of Vestibular Migraine

**DOI:** 10.3389/fneur.2022.944001

**Published:** 2022-07-15

**Authors:** Suming Shi, Dan Wang, Tongli Ren, Wuqing Wang

**Affiliations:** ^1^ENT Institute and Otorhinolaryngology Department, Affiliated Eye and ENT Hospital, Fudan University, Shanghai, China; ^2^NHC Key Laboratory of Hearing Medicine (Fudan University), Shanghai, China

**Keywords:** vestibular migraine, auditory symptoms, hearing loss, tinnitus, aural fullness

## Abstract

**Objectives:**

To investigate the auditory features of patients with vestibular migraine (VM) and to analyze the possible relevant factors of hearing loss.

**Methods:**

A total of 166 patients with VM were enrolled. Demographic variables, age of onset, disease course, distribution of vestibular attacks, characteristics of hearing loss, and the coexistence of related disorders, such as visual aura, familial history, motion sickness, nausea, headache, photophobia, otalgia, tinnitus, aural fullness, and phonophobia, were analyzed and compared.

**Results:**

Patients with VM can manifest otalgia (8.4%), tinnitus (51.8%), aural fullness (41%), and phonophobia (31.9%). Of 166 patients, the prevalence of VMw was 21.1% (*n* = 35). Patients with VMw mainly manifested mild and easily reversible low-frequency hearing loss. The proportions of tinnitus and aural fullness were significantly larger in patients with VMw than that in patients with VMo (*P* < 0.05). The duration of vestibular symptoms was significantly shorter in patients with VMw (*P* < 0.05). However, the age of onset, disease course, gender, frequency of vestibular attacks, the coexistence of visual aura, familial history, motion sickness, nausea, headache, photophobia, otalgia, and phonophobia had no significant difference between the two groups.

**Conclusion:**

Auditory symptoms were common in patients with VM. The hearing loss of VM was characterized by a mild and easily reversible low-frequency hearing loss, accompanied by higher proportions of tinnitus and aural fullness, and a shorter duration of vestibular symptoms compared with patients with VMo.

## Introduction

Vestibular migraine (VM) is one of the most common causes of recurrent vertigo, with a high prevalence of 1~2.7% in individuals. It causes significant morbidity with loss of work hours and is considered to be the seventh most disabling disease worldwide. However, VM remains under-recognized because of the broad spectrum of its manifestations.

Clinicians frequently come across patients with migraine symptoms and dizziness, many other audiovestibular symptoms are associated with migraine, including tinnitus (1), aural fullness (2), otalgia (3), sudden sensorineural hearing loss (SSNHL) (4–6), and fluctuating hearing loss (7). However, the detailed characteristics of auditory symptoms and the factors related to hearing loss are far from clear. The more details found regarding the characteristics of auditory manifestations of VM, the easier the VM is recognized.

Therefore, we reviewed 166 patients with VM to evaluate the auditory symptoms and to analyze the possible relevant factors of hearing loss. Moreover, in consideration of the difficulty of distinguishing VM from the early Meniere's disease (MD), in addition to clinical symptoms, gadolinium (Gd) contrast-enhanced MRI was used to visualize endolymphatic hydrops (ELH) and to exclude early MD and other vestibular disorders in this study (8).

## Methods

### Patients

A total of 166 patients with VM were included from April 2016 to April 2017. The criteria used to enroll patients fulfilled the VM diagnostic criteria formulated by the Committee for Classification of Vestibular Disorders of the Bárány Society (9) (Supplementary Table 1). Systematic history inquiry, neurotologic evaluations, including an electric otoscope, audiometry, tympanometry, and other tests when needed were performed. Other vestibular disorders and headaches were excluded. Moreover, to distinguish VM from other vestibular disorders, especially for MD, Gd contrast-enhanced MRI was conducted for all patients enrolled. The clinical manifestations, including presenting age, onset age, disease course, gender, distribution of vestibular attacks and coexisting visual aura, headache, photophobia, familial history, motion sickness, nausea, otalgia, tinnitus, aural fullness, phonophobia, and hearing loss, were collected and compared. For migraine aura, although many patients report typical visual symptoms, like spreading visual scintillations followed by scotoma, others describe less well-defined symptoms, like visual blurring or distortion. Sometimes, typical symptoms follow an atypical spatial or temporal course. Some aura symptoms like aphasia are also often difficult to distinguish from word-finding difficulty due to cognitive fogging. Moreover, patients with visual symptoms are always accompanied by other aura symptoms. Therefore, the migraine aura enrolled in our study was mainly focused on the visual aura. The distribution of vestibular attacks consists of the frequency of vestibular attacks (within 1 year) and duration of vestibular attacks (most frequent). Ethics approval for this retrospective study was obtained from the Ethical Committees of the Eye, Ear, Nose, and Throat Hospital in Shanghai, China.

### Gd Injection and MRI Acquisition

In total, 166 patients with VM were subjected to the bilateral intratympanic Gd injection (8) or intravenous injection of a double dose (0.4 mL/kg body weight) of Gd-HP-DO3A. Then, MRI was performed 4 h later. For the IV method, all patients underwent an IV injection of a double dose (0.4 mL/kg body weight) of Gd-HP-DO3A; 4 h later, MRI was performed. For the IV method, all scans were performed on a 3T MRI scanner (Verio; Siemens Healthcare, Erlangen, Germany) using a 32-channel phased-array receive-only coil. T2-space and 3D real-IR sequence MRIs were applied for collecting images. The parameters were as follows: voxel size = 0.17 × 0.17 × 0.6 mm; scan time = 15 min and 20 s, repetition time = 6,000 ms, echo time = 181 ms, inversion time = 1,850 ms, slice thickness = 0.6 mm, field of view = 160 × 160 mm, and matrix size = 768 × 768. The patients with MD and other organic lesions were excluded.

### Pure Tone Audiometry Test

Hearing thresholds were tested in all patients at the first visit. The hearing thresholds at low frequency (125, 250, and 500 Hz), medium frequency (1k, 2k Hz), and high frequency (4k, 8k Hz) were evaluated to discover details. All patients conducted a PTA test 1 month later. The threshold below 20 dB HL was considered normal.

### Statistical Analysis

Statistical analyses were performed using SPSS Statistics 17 software (IBM, Chicago, IL, USA) package. Data are presented as x̄ ± SD. The Spearman/Pearson correlation coefficient, the Mann-Whitney *U* test, an independent samples *t*-test, the chi-square test, and Fisher's exact test were used for data analyses. Differences were considered to be statistically significant when *p* < 0.05.

### Ethical Consideration

The institutional review boards of the authors' institutions approved this study.

## Results

### Demographics

The group consisted of 131 (78.9%) women and 35 (21.1%) men, with a visiting age of 42.5 ± 14.9 (6–76) years, onset age of 35.7 ± 14.4 (3–71) years, and a disease course of 81.6 ± 121.4 months. Among 166 individuals, 124 (74.7%) cases were diagnosed as VM, 42 (25.3%) cases were diagnosed as probable VM, 23 (13.9%) cases coexisted with visual aura, 91 (54.8%) cases had a family history of VM, 112 (67.5%) cases had motion sickness, 113 (68.1%) cases had nausea, 69 (41.6%) cases had a headache, 35 (21.1%) cases had hearing loss, 14 (8.4%) cases had otalgia, 86 (51.8%) cases had tinnitus, 68 (41%) cases had aural fullness, 53 (31.9%) cases had phonophobia, and 39 (23.5%) cases had photophobia (Table 1).

**Table 1:** Demographic and clinical characteristics of enrolled patients (*n* = 166).

**Variables**	**Values**
Presenting age, yr, mean±SD	42.5 ± 14.9
Onset age, yr, mean±SD	35.7 ± 14.4
Disease duration, m, mean±SD	81.6 ± 121.4
Gender, *n* (%)	
Female	131 (78.9)
Male	35 (21.1)
Definite VM, *n* (%)	124 (74.7)
Probable VM, *n* (%)	42 (25.3)
Visual aura, *n* (%)	23 (13.9)
Headache, *n* (%)	69 (41.6)
Photophobia, *n* (%)	39 (23.5)
Family history of vertigo, *n* (%)	91 (54.8)
Motion sickness, *n* (%)	112 (67.5)
Nausea, *n* (%)	113 (68.1)
Hearing loss, *n* (%)	35 (21.1)
Unilateral	29 (17.5)
Bilateral	6 (3.6)
Otalgia, *n* (%)	14 (8.4)
Tinnitus, *n* (%)	86 (51.8)
Aural fullness, *n* (%)	68 (41.0)
Phonophobia, *n* (%)	53 (31.9)

As Table 2 shows, there are 77.9% (102/131) female and 22.1% (29/131) male patients with VMo, and 82.9% (29/35) female and 17.1% (6/35) male patients with VMw, with no significant gender difference between the two groups (*P* = 0.520). The onset age of the disease tended to be younger in patients with VMw (31.8 ± 14.1 years) than that in those with VMo (36.8 ± 14.3 years); however, it was not significantly different (*P* = 0.051). The average disease duration from the vestibular onset in patients with VMw (99.2 ± 144.8 months) was longer than the duration in patients with VMo (76.5 ± 113.3 months); however, it was statistically insignificant (*P* = 0.976). The proportions of definite and probable VM were similar between the two groups (*P* = 0.708).

**Table 2:** Clinical features of patients with VM.

	**VMo (*n* = 131)**	**VMw (*n* = 35)**	***P-*value**
Presenting age (y)	43.2 ± 14.8	40.1 ± 15.0	0.244
Onset age (y)	36.8 ± 14.3	31.8 ± 14.1	0.051
Disease course (m)	76.5 ± 113.3	99.2 ± 144.8	0.976
Gender, n (%)			
Female	102 (77.9)	29 (82.9)	0.520
Male	29 (22.1)	6 (17.1)	
Vestibular migraine, *n* (%)			
Definite	97 (74.0)	27 (77.1)	0.708
Probable	34 (26.0)	8 (22.9)	
Visual aura, *n* (%)	16 (12.2)	7 (20.0)	0.236
Headache, *n* (%)	15 (42.9)	54 (41.2)	0.862
Photophobia, *n* (%)	9 (25.7)	30 (22.9)	0.727
Family history of VM, *n* (%)	74 (56.5)	17 (48.6)	0.403
Motion sickness, *n* (%)	90 (68.7)	22 (62.9)	0.512
Nausea, *n* (%)	93 (71.0)	20 (57.1)	0.118
Otalgia, *n* (%)	10 (7.6)	4 (11.4)	0.473
Tinnitus, *n* (%)	58 (44.3)	28 (80.0)	0.000**
Aural fullness, *n* (%)	37 (28.2)	31 (88.6)	0.000**
Phonophobia, *n* (%)	41 (31.3)	12 (34.3)	0.736

VMo, VM without hearing loss; VMw, VM with hearing loss. **p < 0.01.

### Comorbidities

There is no significant difference in the prevalence of coexisting visual aura, headache, photophobia, family history of VM, motion sickness, and nausea between the two groups (*P* > 0.05) (Table 2). Twenty-eight cases (80%) had tinnitus in the VMw group, whereas the number was 58 cases (44.3%) in the VMo group; this difference was significantly different (*P* = 0.000) (Table 2). The proportion of patients with a history of aural fullness was significantly larger in the VMw group (31, 88.6%) than that in the VMo group (37, 28.2%) (*P* = 0.000) (Table 2). Four cases (11.4%) had otalgia in the VMw group, and 10 cases (7.6%) in the VMo group (*P* = 0.473) (Table 2). Twelve (34.3%) cases had phonophobia in the VMo group, and 41 cases (31.3%) in the VMw group (*P* = 0.736) (Table 2).

As Table 3 shows, 92.9% (13/14) of cases had otalgia on one side, 7.1% (1/14) of cases on both sides; 58.1% (50/86) of cases has tinnitus on one side, 41.9% (36/86) of cases on both sides; 79.7% (55/68) of cases had aural fullness on one side, and 20.3% (13/68) of cases on both sides.

**Table 3:** Affected sides of auditory symptoms.

**Symptoms**	**VM, % (n/N)**
Hearing loss	
Unilateral	82.9 (29/35)
Bilateral	17.1 (6/35)
Otalgia	
Unilateral	92.9 (13/14)
Bilateral	7.1 (1/14)
Tinnitus	
Unilateral	58.1 (50/86)
Bilateral	41.9 (36/86)
Aural fullness	
Unilateral	79.7 (55/68)
Bilateral	20.3 (13/68)

*VM, vestibular migraine*.

### Distribution of Vestibular Attacks

The vestibular attacks included vertigo, dizziness, and lightheadedness. The frequency of vestibular attacks and their duration are illustrated in Table 4. No significant difference was noted in the frequency of vestibular attacks between the two groups (*P* = 0.537). The frequency of 1 month-1 year and 1 week-1 month tends to be more common in both groups; the frequency of > 1 year was the least in both groups. The duration of VM was not confined to 5 min-72 h, while some cases can last for <5 min. The duration of vestibular attacks tends to be shorter in the VMw group (VMw, 16/35, 45.7%, <5 min; VMo, 35/131, 26.7%, and <5 min) (*P* = 0.031).

**Table 4:** Distribution of vestibular attacks.

**Attacks, *n* (%)**	**VMo, *n* = 131**	**VMw, *n* = 35**	***P-*value**
Frequency			
<1 day	21 (16.0)	6 (17.1)	0.537
1 day~1 week	15 (11.5)	2 (5.7)	
1 week~ 1 month	26 (19.8)	11 (31.4)	
1 month~1 year	60 (45.8)	13 (37.1)	
>1 year	9 (6.9)	2 (5.7)	
Duration			
<1 min	33 (25.2)	13 (37.1)	0.031*
1 min~5 min	2 (1.5)	3 (8.6)	
5 min~72 h	96 (73.3)	19 (54.3)	

VMo, VM without hearing loss; VMw, VM with hearing loss. *p < 0.05.

### Cochlear Function

The PTA tests were conducted for all patients and repeated 1 month later. Of the 35 patients with hearing loss, 6 cases (17.1%) had bilateral hearing loss, and 29 cases (82.9%) had unilateral hearing loss (Table 3). Twenty-seven cases had low-frequency hearing loss (125, 250, and 500 Hz), 3 cases had low-frequency (125, 250, and 500 Hz) and medium-frequency (1k, 2k Hz) hearing loss, and 5 cases had low-frequency (125, 250, and 500 Hz) and high-frequency (4k, 8k Hz) hearing loss. Within 1 month, hearing loss from low to medium frequency completely transitioned for all patients. The hearing loss at high frequency had no change. Audiograms of a patient with VMw were shown (Supplementary Figure 1).

As shown in Table 5, patients with VMw were mainly manifested with mild- to low-frequency hearing loss. The average hearing thresholds of the patients with VMw were significantly higher than that of the VMo at frequencies of 125, 250, and 500 Hz (*P* = 0.000), while no significant difference was noted at low frequencies and high frequencies between the two groups (*P* > 0.05) (Table 5).

**Table 5:** Auditory thresholds of patients with VM.

**Frequency(Hz)/ Threshold(dB)**	**125**	**250**	**500**	**1k**	**2k**	**4k**	**8k**
VMo	17.5 ± 4.1	17.3 ± 3.6	16.3 ± 3.5	16.2 ± 3.9	16.4 ± 5.4	16.4 ± 5.6	21.5 ± 11.9
VMw	32.5 ± 9.5	34.3 ± 12.2	28.8 ± 11.5	20 ± 9.0	18.3 ± 7.3	16.8 ± 8.5	21.4 ± 12.9
*P-v*alue	0.000**	0.000**	0.000**	0.051	0.192	0.679	0.880

VMo, VM without hearing loss; VMw, VM with hearing loss. **p < 0.01.

### Image Findings

To distinguish VMw from other vestibular disorders and observe the distribution of ELH in patients with VM, all enrolled patients were subjected to the 3T-MRI. Other organic lesions were excluded. All patients (*n* = 166, 100%) had no ELH in both ears, including 131 cases with VMo and 35 cases with VMw.

## Discussion

The study identified the auditory features of patients with VM and analyzed the possible relevant factors for hearing loss. These results can be summarized as follows:

Auditory symptoms were common in patients with VM. The hearing loss of VM was characterized by a mild and easily reversible low-frequency hearing loss, accompanied by a higher proportion of tinnitus and aural fullness, and a shorter duration of the vestibular symptom. Moreover, to ensure the accuracy of the diagnosis of VM, a 3-T MRI was used to exclude other vestibular disorders, including MD at an early stage. No ELH was noted in all patients.

Similar to other subtypes of migraine, VM has a female preponderance, with a reported female-to-male ratio of 1.5~5 to 1, the ratio is 3.7:1 in our study. The median ages of VM symptoms are mid-30–40 s (10–12); our data showed the onset age of 35.7 ± 14.4 (3–71) years, and 2 cases were under 4 years old. VM is the most common vestibular affliction in children (13), its onset may be younger but is often not recognized until children are old enough to properly describe their symptoms. In 41.6% of cases, vestibular and migraine symptoms began concurrently, which was similar to the previous study (42.7%) (14). The proportion of photophobia (23.5%) was lower in our study compared with previous research (15, 16). Apart from the headache, vestibular symptoms, photophobia, and phonophobia, patients with VM may experience visual aura. Visual aura occurred in 13.9% of our patients, it was lower than that observed elsewhere (one-quarter to one-third) (11, 15, 17, 18). Numerous studies over the years have documented familial aggregation of patients with migraine, and it is considered to be a genetic disease (19). Likewise, about 40~90% of patients with VM had a family history, and our result of 54.8% was within the range. Motion sickness is a well-recognized comorbidity in migraine (20). Around two-thirds of patients with migraine have life-long sensitivity to motion sickness and many have spontaneous bouts of motion sickness without exposure to motion. Similarly, motion sickness was endorsed by most of our patients (67.5%). However, motion sickness is also associated with gravitational sensor dysfunction, examination of vestibular-evoked myogenic potentials, and subjective visual vertical would be useful in a further study. Nausea is also a common accompanying symptom of VM; 68.1% of cases coexisted with nausea during VM attacks in our data, and the prevalence can be higher (80.2%) (15).

The VM was considered closely related to auditory symptoms, including hearing loss, tinnitus, aural fullness, and phonophobia. However, the characteristics of cochlear symptoms are far from clear. The incidence of hearing loss in migraine varied from 3.3 to 14% (21–23), and our data were 21.1%, which may indicate that the prevalence of hearing impairment in VM may be higher. A transient and reversible unilateral or bilateral hearing loss during a migraine attack and the intermittent period have been mentioned in some studies (1, 4–6, 24, 25). Our data showed only 6 cases (3.6%) had bilateral hearing loss, and 29 cases (17.5%) had unilateral hearing loss. Mild and reversible low-frequency (125, 250, and 500 Hz) hearing loss was noted in our patients, Fluctuating hearing loss, tinnitus, and aural pressure may occur in vestibular migraine, but the hearing loss does not typically progress to severe hearing loss over the years in VM compared to MD, the auditory features helped distinguish VM from MD (22, 26, 27), idiopathic SSNHL, and other vestibular disorders. It is also worth mentioning that different treatments were applied according to the symptoms of the patients. Patients with apparent incentives and with infrequent and tolerable attacks do not need pharmacological treatment. Acute attacks can be ameliorated in some patients with antiemetic drugs, such as diphenhydramine, meclizine, and metoclopramide. Frequent attacks may warrant pharmacological prophylaxis with metoprolol, amitriptyline, topiramate, valproic acid, or flunarizine. Patients with insomnia, anxiety, or depression were given symptomatic treatment. Therefore, the hearing loss recovered after treatment or spontaneously requires further research. The mechanism of hearing loss remains unclear, several theories have been proposed: (1) Vasospasms associated with migraine in small arterioles within the cochlea and the labyrinth can induce the endolymphatic hydrops (6, 26, 28). However, our study found no cochlear or vestibular hydrops in our patients. To exclude the possibility of overlap of MD and VM, patients with obvious ELH have been excluded. Moreover, it has been reported that the sensitivity of MRI scans is 50% with a different technique and probably less (29), therefore, further studies are needed. (2) Some inflammations and neurotransmitters involved in the pathogenesis of migraine affect the inner ear and the central auditory system (30). (3) A genetic deficiency of ion channels would be related to VM. Furthermore, channels expressed both in the inner ear and in the brain could affect peripheral and central auditory dysfunction (26, 31).

During a migraine attack, not only the auditory system may get damaged and hearing loss may be initiated, but also the central sensitization in the context of migraine will produce excessively high and distorted auditory signals in the central compensation, leading to tinnitus, otalgia, aural fullness, and phonophobia more likely to be perceived (32, 33). The proportion of tinnitus was reported to be 7.5~50% among cochlear symptoms of migraine, our data were similar (51.8%). Patients with classic migraine are more likely to report ear pain than patients with other types of headaches, and patients with otalgia are more likely to report headaches than patients who do not have otalgia (3, 34). However, the proportion of otalgia (8.4%) was much less than tinnitus. The underlying mechanism may be that the sensation in the ear is under the control of afferents from the trigeminal nerve. Moreover, idiopathic SSNHL rarely had otalgia, which may be used to distinguish it from VM with hearing loss. The prevalence of aural fullness and phonophobia were 41 and 31.9%, respectively in our patients. Migraine features were commonly associated with aural fullness (2, 35, 36) and phonophobia, which was also possibly attributed to the activation of the trigeminal nerve and central hypersensitivity. Moreover, aural fullness is correlated with low-frequency hearing loss. However, some patients complaining of aural fullness had no increased hearing thresholds in our study. However, the cochlear function was evaluated only by the PTA test in our study, but an objective way of demonstrating cochlear function would have been otoacoustic emissions.

To explore the clinically related factors of hearing loss, we compared the gender, onset age, disease course, visual aura, motion sickness, family history, headache, photophobia, otalgia, tinnitus, aural fullness, phonophobia, and frequency and duration of vestibular attacks between patients with and without hearing loss. The onset age of the vestibular attacks tends to be younger in patients with VMw (31.8 ± 14.1 years) than that in patients with VMo (36.8 ± 14.3 years); however, it was not significantly different (*P* = 0.051). Further studies are needed to verify this point. The proportion of tinnitus was significantly higher in the VMw group than that of the VMo group. On one hand, it is widely accepted that tinnitus is initiated by hearing loss that subsequently causes abnormal hyperexcitability and neural synchronization in the central auditory system. On the other hand, during a migraine attack, not only the auditory system may be damaged and acute tinnitus may be initiated but also the central sensitization in the context of migraine will produce excessively high and distorted auditory signals in the central compensation (32, 33), which is more likely to be perceived. The higher prevalence of aural fullness in the VMw group may also be a result of the low-frequency hearing loss and the central sensitization. Unilateral auditory symptoms were predominant in the 166 patients with VM (5).

The frequency of vestibular attacks has individual variations, and frequencies of 1 week~1 month and 1 month~1 year were common in two groups. However, no evidence indicated that frequent vestibular attacks were correlated with hearing loss. The most frequent duration of vestibular attacks was minutes to hours (37), the percentage of seconds to ~5 min in the VMw group was significantly higher than that in the VMo group. However, the underlying mechanism still needs exploration. We speculated that the vestibular function in VM with hearing loss might be more sensitive to tiny movement, which can manifest as transient attacks. Furthermore, similar to cochlear migraine proposed by several scholars (7), the cochlear system might be preferentially affected, causing a hearing loss but with a quick vestibular recovery. Further studies and more comprehensive vestibular function tests were needed.

Our research still leaves much to be desired. First, other auditory tests have not been performed except PTA. Second, patients with VM have individual variations, the proposed clinical diagnostic criteria of VM were not able to capture all patients, including patients with cochlear migraine (7). Third, vestibular functions were not analyzed. Moreover, migraine and MD might be inherited as a symptom cluster (38), and distinguishing VM from MD is still difficult. Therefore, it still needs further research.

## Conclusion

In conclusion, auditory symptoms, including tinnitus, aural fullness, phonophobia, and otalgia were common in patients with VM. The hearing impairment of patients with VM was mainly manifested as low frequency and easily reversible hearing loss. In patients with VMw, they were accompanied by a higher proportion of tinnitus and aural fullness and a shorter duration of vestibular attacks. To explore the correlated clinical factors of hearing loss, further studies are warranted.

## Data Availability Statement

The original contributions presented in the study are included in the article/Supplementary Material, further inquiries can be directed to the corresponding author.

## Ethics Statement

Ethics approval for this retrospective study was obtained from the Ethical Committees of the Eye, Ear, Nose, & Throat Hospital in Shanghai, China. Written informed consent to participate in this study was provided by the participants' legal guardian/next of kin.

## Author Contributions

SS: drafting the manuscript. SS and DW: analysis and/or interpretation of data. SS, DW, and TR: acquisition of data. WW: design of study and revising the manuscript critically for important intellectual content. All authors contributed to the article and approved the submitted version.

## Funding

This study was supported by the National Natural Science Foundation of China (No. 82101222) and the Natural Science Foundation of Shanghai (No. 20ZR1409600).

## Conflict of Interest

The authors declare that the research was conducted in the absence of any commercial or financial relationships that could be construed as a potential conflict of interest.

## Publisher's Note

All claims expressed in this article are solely those of the authors and do not necessarily represent those of their affiliated organizations, or those of the publisher, the editors and the reviewers. Any product that may be evaluated in this article, or claim that may be made by its manufacturer, is not guaranteed or endorsed by the publisher.
