# Supplementary figures and images for: Auditory Manifestations of Vestibular Migraine
Source: Front Neurol. 2022 Jul 15;13:944001. doi: 10.3389/fneur.2022.944001 (PMC9334870; doi:10.3389/fneur.2022.944001)

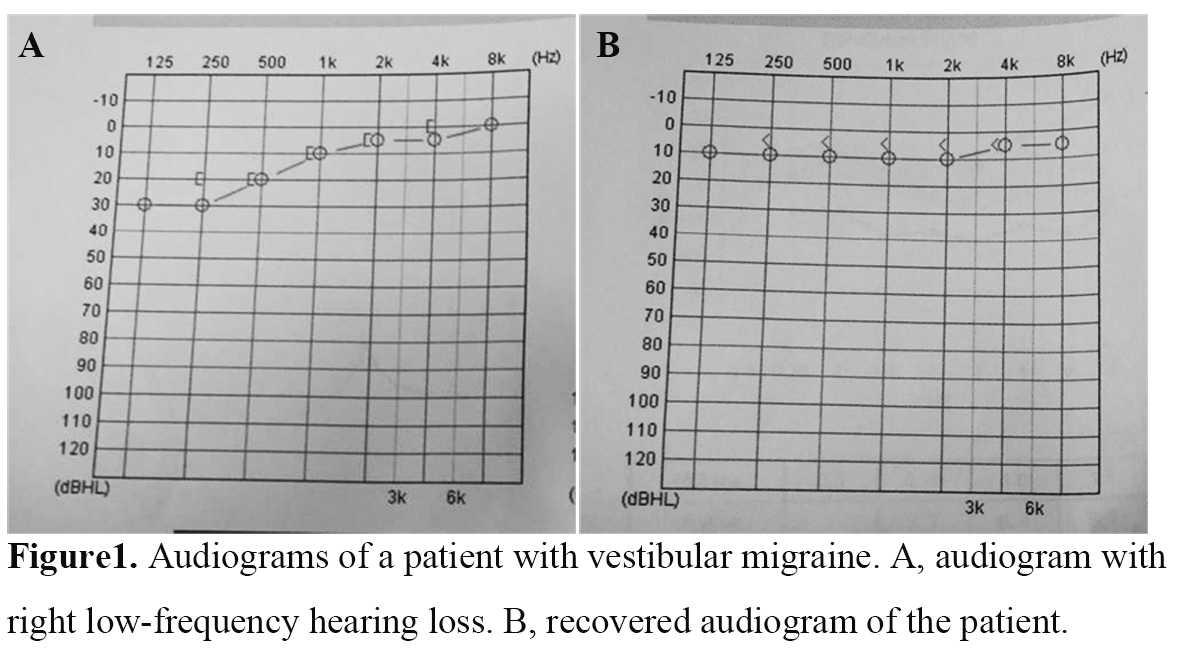

Supplement: Supplementary file 1 [file Image_1.tif]
