# Supplementary material for: Auditory Manifestations of Vestibular Migraine
Source: Front Neurol. 2022 Jul 15;13:944001. doi: 10.3389/fneur.2022.944001 (PMC9334870; doi:10.3389/fneur.2022.944001)
Supplement: Supplementary file 2 [file Table_1.docx]

TABLE 1. Diagnostic criteria of vestibular migraine.

| **1. Vestibular migraine**  A. At least 5 episodes with vestibular symptoms of moderate or severe intensity, lasting 5 min to 72 hours. |
| --- |
| B. Current or previous history of migraine with or without aura according to the International Classification of Headache Disorders (ICHD3). |
| C. One or more migraine features with at least 50% of the vestibular episodes:  –headache with at least two of the following characteristics: one sided location, pulsating quality, moderate or severe pain intensity, aggravation by routine physical activity;  –photophobia and phonophobia;  –visual aura. |
| D. Not better accounted for by another vestibular or ICHD diagnosis.  **2. Probable vestibular migraine**  A. At least 5 episodes with vestibular symptoms1 of moderate or severe intensity, lasting 5 min to 72 hours.  B. Only one of the criteria B and C for vestibular migraine is fulfilled (migraine history or migraine features during the episode)  C. Not better accounted for by another vestibular or ICHD diagnosis |

|  |
| --- |
